# Yiqi Qingre Gao alleviates renal fibrosis in UUO mice via PI3K/AKT pathway

**DOI:** 10.3389/fphar.2025.1538061

**Published:** 2025-03-24

**Authors:** Qi Jin, Qian Li, Liping Yang, Fang Ma, Huimin Mao, Yuyang Wang, Tongtong Liu, Liang Peng, Ping Li, Yongli Zhan

**Affiliations:** ^1^ Guang’anmen Hospital, China Academy of Chinese Medical Sciences, Beijing, China; ^2^ Qinghai Provincial Hospital of Traditional Chinese Medicine, Xining, China; ^3^ Institute of Clinical Medical Sciences, China-Japan Friendship Hospital, Beijing, China

**Keywords:** Yiqi Qingre Gao, chronic kidney diseases, renal fibrosis, PI3K/AKT, network pharmacology

## Abstract

**Introduction:** Renal fibrosis is an endpoint event of various progressive chronic kidney diseases (CKD), but there are no effective antifibrotic treatments. Yiqi Qingre Gao (YQQRG) has shown potential in alleviating CKD, although its exact mechanism of action remains uncertain. This study aims to evaluate the impact of YQQRG on renal fibrosis and to explore the molecular pathways involved.

**Methods:** The study employed a unilateral ureteral obstruction (UUO) mouse model, followed by a 2-week course of YQQRG treatment. Renal function was assessed through measurements of serum creatinine (SCr) and blood urea nitrogen (BUN). Kidneys were collected for histological and molecular biology analysis. To identify the detailed mechanisms, network pharmacology, RNA sequencing (RNA-Seq), transforming growth factor-beta1 (TGF-β1)-stimulated human renal proximal tubular epithelial (HK-2) cells, and molecular docking were used.

**Results:** YQQRG treatment significantly improved renal function, pathological damage, and renal fibrosis in UUO mice. Ten blood-entering components and 403 potential targets of YQQRG were identified by liquid chromatography-mass spectrometry (LC-MS) and network pharmacology. 20,107 targets of renal fibrosis were revealed by RNA-Seq of kidneys from the control and UUO groups. The results of the KEGG pathway enrichment analysis of YQQRG and renal fibrosis were combined, which showed that YQQRG’s renoprotective effects were strongly associated with the phosphatidylinositol 3-kinase (PI3K)/protein kinase B (AKT) signaling pathway. Experimental validation further confirmed that YQQRG suppressed the PI3K/AKT pathway in the renal tissues of UUO mice; the addition of the PI3K/AKT agonist reversed the antifibrotic effects of YQQRG in TGF-β1-stimulated HK-2 cells. Furthermore, molecular docking indicated that YQQRG’s primary active components exhibited a strong binding affinity to critical targets.

**Discussion:** This study initially demonstrated that YQQRG improved renal function and kidney injury in UUO mice by revealing its antifibrotic mechanism, and it operates through the inhibition of the PI3K/AKT pathway, which highlights YQQRG as a potential therapeutic option for treating CKD.

## Graphical Abstract

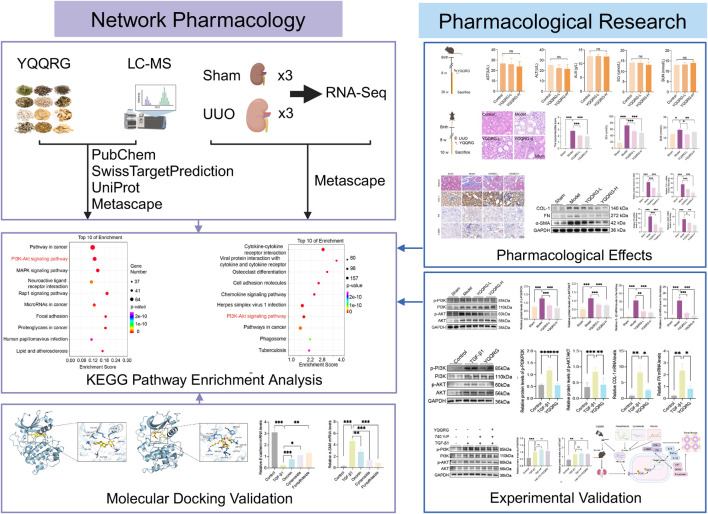

## Highlights

YQQRG largely improved renal function in mice with UUO.
PI^3^K/AKT signaling pathway may be involved in the treatment of UUO.
YQQRG prevented PI3K/AKT signaling pathway in mice with UUO.
These data provide evidence that YQQRG is a promising therapeutic intervention for the management of UUO.

## Introduction

The incidence of Chronic Kidney Disease (CKD) is on a continuous upward trend with the aging of the global population and the rising incidence of cardiovascular diseases (Kidney Disease: Improving Global Outcomes KDIGO CKD Work Group, 2024). Renal fibrosis is considered to be the end event of various CKDs (Yamashita and Kramann, 2024), and has become a key therapeutic target in the management of CKD (Liu, 2024). Current therapeutic approaches focus primarily on slowing disease progression and controlling complications and include renin-angiotensin system inhibition and renal replacement therapy. However, these strategies are often associated with adverse effects that affect patients’ quality of life (Levin et al., 2024). Therefore, new therapeutic interventions are urgently needed to reduce renal fibrosis.

Traditional Chinese Medicine (TCM) has demonstrated significant potential in CKD management, especially in slowing down the progression of renal fibrosis and improving patients’ quality of life, which has received increasing clinical recognition (Zhong et al., 2019; Zhu et al., 2023). Multi-component and multi-targeted therapies of TCM can have synergistic effects with Western medicine, especially in terms of personalized treatment strategies and reduction of adverse effects (Deng et al., 2023). Therefore, recent studies have gradually elucidated the efficacy mechanisms of TCM in the treatment of CKD and renal fibrosis (Huang M. et al., 2023; Yong et al., 2023).

Yiqi Qingre Gao (YQQRG), a TCM compound formula with over two decades of clinical application, comprises 12 herbal components, including *Astragalus Radix* (Huangqi), *Atractylodes Macrocephala Koidz* (Baizhu), *Dioscorea nipponica Makino* (Chuanshanlong). Clinical studies have shown that YQQRG can effectively reduce 24-h urine protein level, serum total cholesterol and urine erythrocyte count in patients with chronic glomerulonephritis, as well as increase the levels of albumin and immunoglobulin (IgA, IgG) (Zhan and Dai, 2003). Experimental studies further revealed the ability of YQQRG to repair podocyte damage (Zhan et al., 2014), inhibit mesangial cell proliferation (Yang et al., 2016), and improve kidney function in mice models. Notably, Dioscin, a major component of Dioscorea nipponica Makino (Chuanshanlong), has been shown to ameliorate renal fibrosis through nuclear factor-kappaB (NF-κB) signaling pathway inhibition, as evidenced by reduced expression of inflammatory cytokines (interleukin-1 beta (IL-1β), IL-6, IL-18, tumor necrosis factor-alpha (TNF-α), and decreased NF-κB p65 phosphorylation (Wang et al., 2023). However, the effects of YQQRG on renal tubular epithelial cells remain underexplored.

This investigation employs a comprehensive methodological approach, including liquid chromatography-mass spectrometry (LC/MS) analysis of YQQRG components, network pharmacology, RNA sequencing (RNA-Seq), *in vitro* and *in vivo* experiments, and molecular docking studies. This multifaceted approach aims to elucidate the mechanisms underlying YQQRG’s anti-fibrotic effects, thereby providing a scientific foundation for its clinical application in renal fibrosis management.

## Material and methods

### Preparation of YQQRG

Composition of YQQRG: *Astragalus Radix* (Huangqi), *Atractylodes Macrocephala Koidz* (Baizhu), *Radix Saposhnikoviae* (Fangfeng), *Lonicerae Japonicae Flos* (Jinyinghua), *Fructus Forsythiae* (Lianqiao), *Herba Duchesnea Indica* (Shemei), *Herba Hedyotis* (Baihuasheshecao), *Poria* (Fulin), *Rhizoma Alismatis* (Zexie), *Herba Leonuri* (Yimucao), *Rhizoma Imperatae* (Baimaogen), *Dioscorea nipponica Makino* (Chuanshanlong). The preparation process: decoction and extraction, concentration, alcohol precipitation, recovery of ethanol, water precipitation, filtration, supernatant liquid dispensing, canning, disinfection, packaging. The final product, with a concentration of 4.5 g of raw herbs per milliliter, is packaged in 250 g bottles and stored at 4°C. All procedures are conducted under strict quality control by the Pharmacy Department of Guang’anmen Hospital, China Academy of Chinese Medical Sciences (Approval No: 98 JEWIM PHARMA 058).

### Dosage determination and administration

Based on previous experimental data and dose conversion principles, two dosage levels were selected to reflect clinically relevant concentrations while ensuring safety and efficacy in animal models. The low dose (2.9 g/kg, equivalent to 10 times the human dosage) and high dose (5.8 g/kg, equivalent to 20 times the human dosage) were chosen to evaluate dose-dependent effects. For administration, the formulation was diluted with saline to achieve the required concentrations, as previously described (Zhan et al., 2014; Wen et al., 2015).

### Animals

Specific pathogen free male mice were obtained from Cyagen (License No. SCXK (SU) 2022-0016) and housed at the Institute of Clinical Medical Sciences, China-Japan Friendship Hospital. The animals were maintained under controlled environmental conditions, including a temperature range of 17–24°C, a 12-h light/dark cycle, and 50%–60% relative humidity, with *ad libitum* access to standard rodent chow and water. All experimental procedures were conducted in compliance with the institutional ethical guidelines for the care and use of laboratory animals and were approved by the ethics committee of the Institute (Approval No. ZRDWLL0056). All mice had a 7-day adaptation period before experiment.

### Modeling and treatment

Mice were randomly allocated into two groups: a sham surgery group and a unilateral ureteral obstruction (UUO) model group. Anesthesia was induced using 1% pentobarbital sodium (0.1 mL/10 g body weight). The UUO group underwent surgical ligation of the left ureter with a 4-0 silk/prolene suture to induce renal fibrosis, while the sham group underwent an identical surgical procedure without ureteral ligation. Postoperatively, 0.2 mL of antibiotic was administered intraperitoneally to prevent infection, the incision was sutured, and erythromycin ointment was applied to the wound. The UUO model was well-tolerated, with no mortality observed during the surgical procedure or the subsequent 2-week treatment period.

Safety experiment: To evaluate the safety profile of YQQRG, control mice were administered low (2.9 g/kg/d) and high (5.8 g/kg/d) doses of YQQRG via oral gavage once daily for 12 weeks (*n* = 6 per group).

Validity experiment: A total of 24 mice, including 18 successfully modeled UUO mice and 6 sham-operated mice, were divided into four groups: sham-operated, UUO model, low-dose YQQRG (YQQRG-L, 2.9 g/kg/d), and high-dose YQQRG (YQQRG-H, 5.8 g/kg/d). Treatments were initiated 24 h post-surgery and administered orally once daily for 2 weeks. The sham-operated group were administered an identical volume of sterile physiological saline (0.9% NaCl) through the same route of administration, as control group (*n* = 6 per group).

### Determination of liver index and kidney index

Twenty-four hours after the final administration, mice were euthanized, and body weights were recorded. The liver and kidneys were carefully excised, and any surrounding adipose tissue or fascia was removed. Organs were rinsed with saline, blotted dry on filter paper, and weighed. The liver index and kidney index were calculated as the ratio of liver or kidney weight to body weight, respectively.

### Serum biomarker analysis

Blood samples were collected via cardiac puncture and centrifuged at 3,500 rpm for 10 min to isolate serum. The concentrations of blood urea nitrogen (BUN), serum creatinine (SCr), alanine aminotransferase (ALT), aspartate aminotransferase (AST), and albumin (ALB) were quantified using commercially available reagent kits (Nanjing Jiancheng Institute of Biological Engineering, Nanjing, China) according to the manufacturer’s protocols.

### Histopathological evaluation of liver and kidney tissues

Liver and Kidney were similarly fixed in 4% paraformaldehyde, dehydrated, permeabilized, and embedded in paraffin. Sections of 4 µm thickness were cut, deparaffinized, rehydrated, and stained with either H&E or Masson’s trichrome to assess structural changes and fibrosis.

The tubulointerstitial injury score: Renal histopathological assessment was conducted by evaluating the severity of tubular lesions, including tubular dilatation, atrophy, interstitial inflammation, and fibrosis. A semi-quantitative scoring system was applied as follows: 0 points for no lesions; 1 point for lesions affecting <25% of the tissue; 2 points for 25%–50% involvement; 3 points for 50%–75% involvement; and 4 points for >75% involvement. Each specimen was examined under a light microscope at a scale bar of 50 μm, and 10 randomly selected fields were analyzed to calculate the mean renal tubular injury score.

### Immunohistochemistry (IHC) analysis

Paraffin sections (3 µm thick) were dewaxed, antigenically repaired with EDTA antigen retrieval solution, blocked using immunostaining blocking solution, and incubated overnight at 4°C with the following primary antibodies: alpha-smooth muscle actin (ɑ-SMA) (Boster, BM0002, 1:50), fibronectin (FN) (Proteintech, 15613-1-AP, 1:50), and collagen type I (COL-1) (Proteintech, 14695-1-AP, 1:50),p-PI3K (Proteintech, 60225-1, 1:50), p-AKT (Proteintech, 66444-1-Ig, 1:50). The sections were then incubated with a secondary antibody for 30 min at room temperature. DAB staining (ZSGB-BIO, ZLI-9018) was used for color development, and hematoxylin was applied to restain the nuclei. Sections were rehydrated and sealed with a neutral mounting medium. Ten random cortical fields per sample were imaged at a scale bar of 50 μm, and the percentage of positively stained areas was quantified using ImageJ software.

### Western blot analysis

Kidney tissues and cells were lysed using RIPA lysis buffer (Beyotime, P0013C) with PMSF (Beyotime, ST506). Protein concentrations were measured with the BCA Protein Assay Kit (Beyotime, P0012S). Equal amounts of protein samples were separated on 10% SDS-PAGE (enzyme, PG112) and transferred onto a PVDF membrane (Millipore, PVH00010). The membrane was blocked with 5% skimmed milk for 2 hours at room temperature, followed by overnight incubation at 4°C with the following primary antibodies: ɑ-SMA (Boster, BM0002, 1:1000), FN (Proteintech, 15613-1-AP, 1:1000), COL-1 (Proteintech, 14695-1-AP, 1:1000), phosphoinositide 3-kinase (PI3K) (Proteintech, 20584-1-AP, 1:1000), p-PI3K (Proteintech, 60225-1, 1:1000), protein kinase B (AKT) (Proteintech, 10176-2-AP, 1:1000), and p-AKT (Proteintech, 66444-1-Ig, 1:1000). After primary antibody incubation, membranes were washed and incubated with appropriate horseradish peroxidase-conjugated secondary antibodies for 2 h at room temperature. Following three washes, protein bands were visualized using enhanced chemiluminescence (Yeasen, 36208ES60), and densitometric analysis was performed using ImageJ software.

### RNA extraction and quantitative real-time PCR (qRT-PCR) detection

Total RNA was extracted from kidney tissues or cells using TRIzol reagent (Invitrogen, 15596018CN). cDNA was synthesized from 1 µg of RNA using the HiScript III All-in-one RT SuperMix for qPCR (Vazyme, R333-01). Quantitative real-time PCR was conducted using a Thermo Fisher (ABI) instrument and Taq Pro Universal SYBR qPCR Master Mix (Vazyme, Q712). Relative mRNA expression levels were calculated using the 2^−ΔΔCT^ method, with β-actin serving as the internal control for normalization. Gene primers were synthesized by BGI (Shenzhen, China) and are listed in Supplementary Table S1.

### Acquisition of blood-entering components and corresponding targets of YQQRG

In the preliminary phase of this study, our research team employed LC-MS to identify 24 bioactive components of YQQRG. Among these, 10 components were detectable in serum and were selected for further analysis. The molecular structures and Canonical SMILES numbers of these components were retrieved from the PubChem database (https://pubchem.ncbi.nlm.nih.gov). The SMILES notations were subsequently imported into the Swiss Target Prediction database (http://www.swisstargetprediction.ch) to predict potential molecular targets. Targets with a probability score >0 were retained, and duplicates were removed. The predicted targets were further validated and standardized using the UniProt database (https://www.uniprot.org).

### LC-MS technology

#### Laboratory instruments

The analysis was performed using an Ultimate 3000 ultra-high performance liquid chromatograph (Thermo Fisher) coupled with an LTQ Orbitrap Velos Pro mass spectrometer (Thermo Fisher). Data acquisition and processing were conducted using the Xcalibur 2.1 workstation (Thermo Fisher). Ultrapure water was prepared using a Milli-Q ultrapure water system (Millipore), and samples were lyophilized using an LGJ-10B freeze dryer (Beijing Sihuan Scientific Instrument Factory).

#### Chromatographic conditions

Chromatographic separation was achieved using a BEH HSS T3 column (2.1 mm × 100 mm, 1.8 μm, Waters Corp.). The mobile phase consisted of acetonitrile (A) (Thermo Fisher, 186350) and 0.1% formic acid aqueous solution (B) (Sigma, 540-69-2) with the following gradient elution program: 0–30 min, 5%–95% A; 30–35 min, 95% A; 35–35.1 min, 95%–5% A; 35.1–40 min, 5% A. The flow rate was maintained at 0.30 mL/min, the column temperature was set at 35°C, and the injection volume was 1 μL.

#### Mass spectrometry conditions

Electrospray ionization source, positive and negative ion modes were scanned separately. Positive ion mode was performed at a capillary temperature of 350°C, a capillary voltage of 35 V, a spray voltage of 3.4 kV, a sheath gas (N_2_) flow rate of 35 psi, and an auxiliary gas (N_2_) flow rate of 10 psi, while negative ion mode was performed at a capillary temperature of 350°C, a capillary voltage of −35 V, a spray voltage of −3.4 kV, a sheath gas (N_2_) flow rate of 35 psi, and an auxiliary gas (N_2_) flow rate of 10 psi. The primary mass spectra of the samples were fully scanned in FT mode (resolution R of 30,000, m/z scanning range of 50–2,000), and the secondary and tertiary mass spectra were scanned using data-dependent scanning. The mass axis calibration of the mass spectra was performed by the external standard method (mass error <5 ppm), with calibration masses of 74.0964, 195.0846, 262.6361, 524.2650, and 1,022.0034 selected for positive ions, and 230.1017, 249.1530, and 407.2803 selected for negative ions.The data were acquired and analyzed using Xcalibur, Metworks and Mass Frontier 7.0 software.

### RNA-seq and data analysis

Total RNA was extracted from kidney tissues using TRIzol reagent (Invitrogen, 15596018CN). RNA libraries were prepared and pooled, followed by sequencing on the Illumina platform. Raw sequencing reads were processed using FAST software to remove low-quality sequences and rRNA contamination. Clean reads were aligned to the reference genome, and differential gene expression analysis was performed using DESeq2 software. Genes with a *p*-value <0.05 and a Log2 fold change ≥2 were considered significantly differentially expressed.

### Kyoto Encyclopedia of Genes and Genomes (KEGG) pathway enrichment analysis

Functional enrichment analysis of target genes was conducted using the Metascape online database (https://metascape.org/gp/index.html), including KEGG pathway analysis. Enriched pathways were ranked by *p*-value, and the top 10 most significant pathways were selected for further investigation.

#### Molecular docking

The three-dimensional structures of the compounds were retrieved from the PubChem database (https://pubchem.ncbi.nlm.nih.gov/) and converted from SDF to PDB format using Open Babel 2.3.2 software. Receptor protein structures were obtained from the Protein Data Bank (https://www.rcsb.org). PYMOL 2.3.4 software was utilized to remove water molecules and ligands from the receptor proteins. The receptor proteins were then hydrogenated and charge-balanced using Autodocktools software, and both receptor proteins and ligand molecules were converted into pdbqt format. Molecular docking was performed using AutoDock Vina 1.1.2, and the docking results were analyzed using the Protein-Ligand Interaction Profiler (PLIP). Visualization of the docking results was conducted using PYMOL.

### Cell culture and treatment

Human renal proximal tubular epithelial (HK-2) cells were cultured in DMEM/F12 complete medium (Gibco, 21331020) supplemented with 10% fetal bovine serum (Gibco, A5256701) and 1% antibiotics (Gibco, 15140122). The optimal concentration of YQQRG was determined using the Cell Counting Kit-8 (DOJINDO, CK04). Cells were treated with transforming growth factor-β1 (TGF-β1) (PeproTech, 100-21) at a concentration of 10 ng/mL and 10% YQQRG medicated serum, which was identified as the optimal concentration. The experimental groups included: (1) control group, (2) TGF-β1 group, (3) YQQRG group, (4) Forsythiaside group (MCE, HY-N0028, 30 µM), (5) Cynaroside group (MCE, HY-N0540, 30 µM), and (6) Ononin group (MCE, HY-N0270, 30 µM), with all treatments lasting 24 h. Additionally, the PI3K/AKT agonist 740Y-P (MCE, HY-P0175A) was used at a concentration of 20 µM. Subgroups included: (1) control group, (2) TGF-β1 group, (3) TGF-β1 + 740Y-P group, and (4) TGF-β1 + 740Y-P + YQQRG group.

### Statistical analysis

All data are presented as mean ± standard error of the mean Statistical analyses and graphical representations were performed using GraphPad Prism software (version 9.5.1) and Adobe Illustrator (version 2022). For normally distributed data, one-way analysis of variance (ANOVA) was conducted, followed by Tukey’s *post hoc* test for multiple comparisons. For non-normally distributed data, the Kruskal–Wallis test was applied, followed by the Wilcoxon rank-sum test. A *p*-value of less than 0.05 was considered statistically significant.

## Results

### YQQRG alleviated kidney injury in UUO mice

**FIGURE 1:**
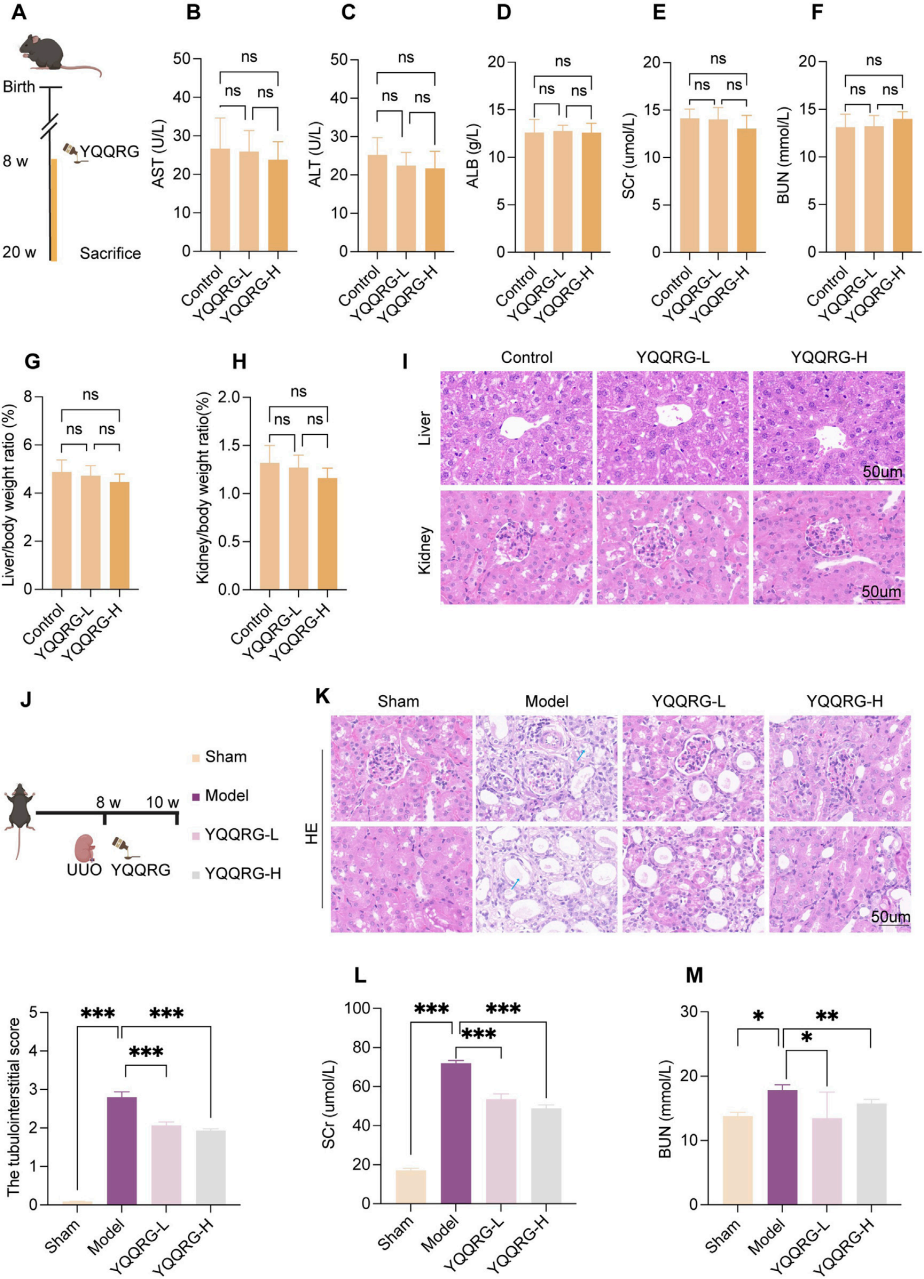
YQQRG relieved kidney injury in UUO mice. **(A)** Safety experimental schedule (*n* = 6). **(B)** AST in different dose groups of control mice. **(C)** ALT in different dose groups of control mice. **(D)** ALB in different dose groups of control mice. **(E)** SCr in different dose groups of control mice. **(F)** BUN in different dose groups of control mice. **(G)** Liver/body weight ratio in different dose groups of control mice. **(H)** Kidney/body weight ratio in different dose groups of control mice. **(I)** Representative HE-stained liver and kidney histological images in different dose groups of control mice. **(J)** Validity experimental schedule (*n* = 6). **(K)** Representative HE-stained kidney histological images in mice after administration. **(L)** Changes in SCr in mice after administration. **(M)** Changes in BUN in mice after administration.

To assess the safety profile of YQQRG (Figure 1A), control mice were administered varying doses of YQQRG. Serum levels of AST, ALT, ALB, SCr, and BUN, as well as liver-to-body weight and kidney-to-body weight ratios, were measured. Histological examination of liver and kidney tissues via HE staining revealed no significant differences, confirming the safety of YQQRG (Figures 1B–I). To evaluate the therapeutic efficacy of YQQRG, a 2-week intervention was administered to UUO model mice (Figure 1J). HE staining showed that there are no significant pathological changes in the renal tissue of mice in the sham group. The kidneys of mice in the UUO model group showed significant tubular damage, including tubular dilatation and atrophy (blue arrow). The tubular damage was ameliorated after the YQQRG intervention (Figure 1K). The results showed that SCr and BUN were significantly elevated in the UUO model group compared to the sham group, and treatment with YQQRG significantly reduced SCr and BUN compared to the UUO model group (Figures 1L,M). These findings suggest that UUO mice suffer from substantial renal dysfunction, which can be effectively alleviated by YQQRG treatment.

### YQQRG improved renal fibrosis in UUO mice

**FIGURE 2:**
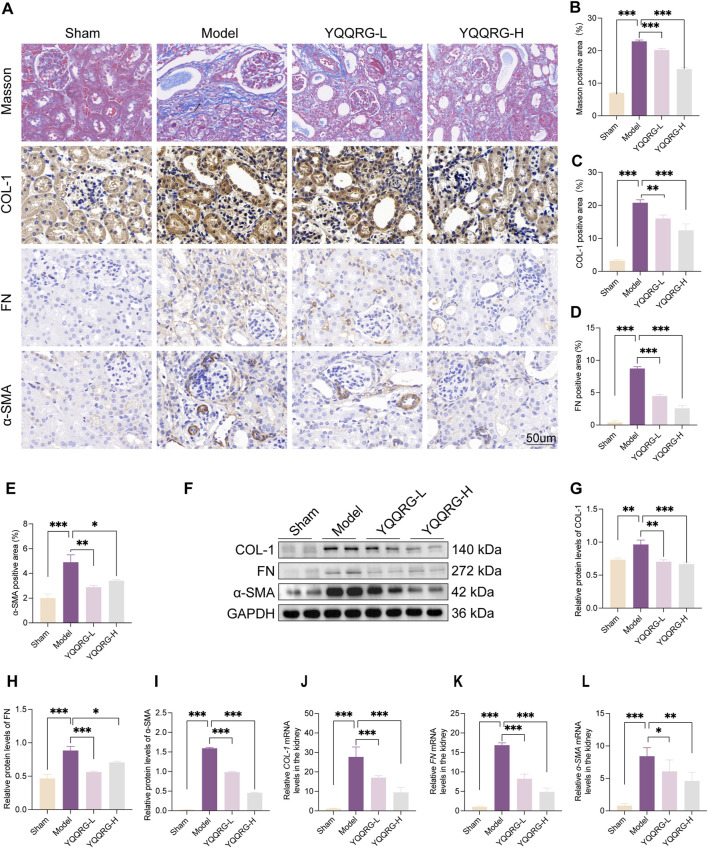
YQQRG improved renal fibrosis in UUO mice. **(A)** Representative images of Masson, IHC of COL-1, FN, ɑ-SMA in each group. **(B)** The fibrosis area on Masson staining. **(C)** COL-1 positive area. **(D)** FN positive area. **(E)** ɑ-SMA positive area. **(F)** Representative Western blot of COL-1, FN, ɑ-SMA in each group. **(G)** Relative expression of COL-1/GAPDH. **(H)** Relative expression of FN/GAPDH. **(I)** Relative expression of ɑ-SMA/GAPDH. **(J)** The relative mRNA levels of *COL-1*. **(K)** The relative mRNA levels of *FN*. **(L)** The relative mRNA levels of *ɑ-SMA*.

Masson’s trichrome and IHC staining revealed significant collagen deposition around renal tubules and interstitium in the kidneys of UUO model mice (indicated by black arrows), which was markedly reduced following YQQRG intervention. Furthermore, the expression levels of fibrosis-related markers, including COL-1, FN, and α-SMA, were significantly elevated in the kidneys of UUO model mice but were notably downregulated after YQQRG administration (Figures 2A–E). Western blot analysis and quantitative assessment further confirmed these findings, demonstrating significantly higher expression levels of COL-1, FN, and α-SMA in the UUO model group compared to the sham-operated group. Conversely, YQQRG treatment significantly reduced the expression of these markers in kidney tissues (Figures 2F–I). Additionally, mRNA levels of COL-1, FN, and α-SMA were elevated in the UUO model group, and YQQRG treatment effectively mitigated these increases (Figures 2J–L). These results collectively indicate that YQQRG exerts a therapeutic effect by ameliorating renal fibrosis in UUO mice.

### KEGG pathway enrichment analysis

**FIGURE 3:**
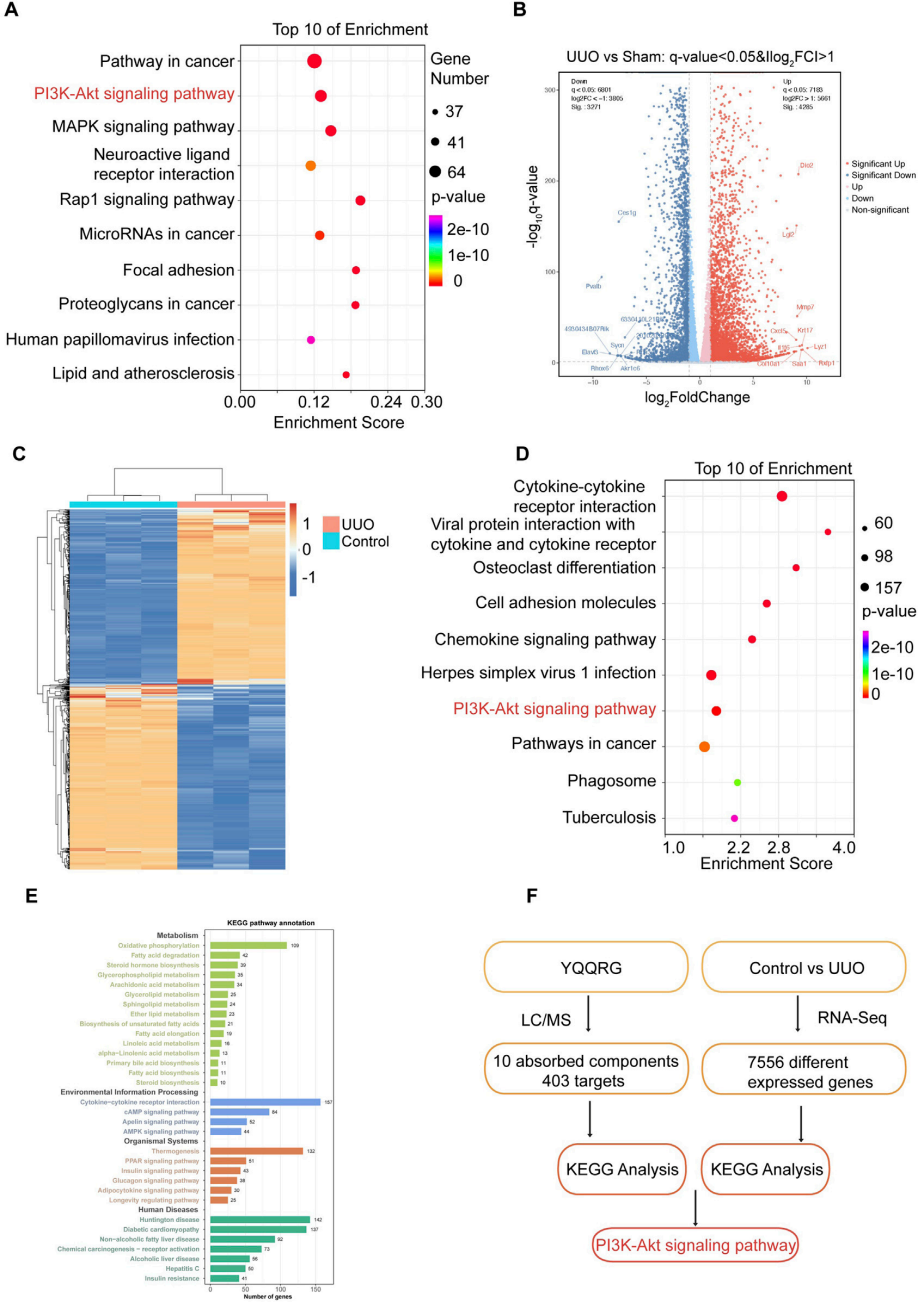
The Screening of PI3K/AKT signaling pathway. **(A)** The top 10 KEGG signaling pathways of targets of YQQRG are shown; the X-axis indicates the enrichment score, the bubble size indicates the gene numbers, and the color indicates the *p* value10 active components of YQQRG. **(B)** Volcano plot of differently expressed genes between the control group and UUO group. **(C)** Heat map of differently expressed genes between the control group and UUO group. **(D)** Bubble diagram of KEGG analysis of different genes (upregulated in UUO group). **(E)** Clustering analysis of KEGG analysis of different genes. **(F)** Screening Flowchart.

In our previous study, 24 components of YQQRG were identified using LC-MS technology, of which 10 blood-entering components were selected for further investigation (Table 1). A total of 403 potential targets associated with these 10 components were identified using the PubChem, Swiss Target Prediction, and UniProt databases. Functional enrichment analysis of these targets was performed using the Metascape online tool, and the top 10 enriched KEGG pathways were selected (Figure 3A). To explore the molecular mechanisms underlying renal fibrosis, RNA-seq transcriptome sequencing was conducted, identifying 20,107 genes, of which 7,556 were significantly differentially expressed (4,285 upregulated and 3,271 downregulated) (Figures 3B,C). KEGG pathway enrichment analysis of the upregulated genes revealed the top 10 enriched pathways (Figures 3D,E). By integrating the KEGG pathway results from YQQRG targets and renal fibrosis-related genes, the PI3K/AKT signaling pathway emerged as a top-ranked pathway, suggesting its potential role as a key anti-fibrotic mechanism of YQQRG (Figure 3F).

**TABLE 1:** YQQRG components that absorbed in serum by LC-MS technique.

Compound	CAS	PubChem CID	Molecular formula	Structure	Source
Neochlorogenic acid	906-33-2	5280633	C_16_H_18_O_9_	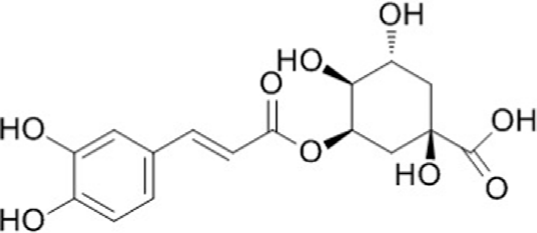	Jinyinghua
Cryptochlorogenic acid	905-99-7	9798666	C_16_H_18_O_9_	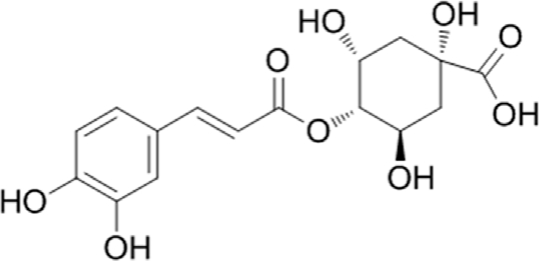	Jinyinghua
Chlorogenic acid	327-97-9	1794427	C_16_H_18_O_9_	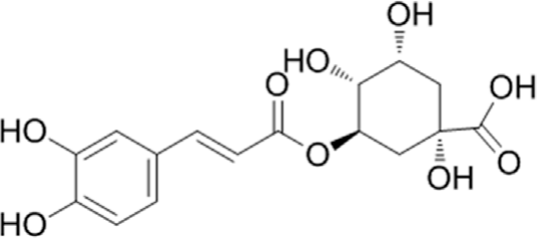	Jinyinghua
Acteoside	61276-17-3	5281800	C_29_H_36_O_15_	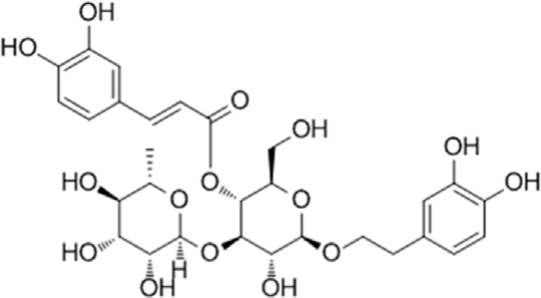	Lianqiao
Forsythiaside	79916-77-1	5281773	C_29_H_36_O_15_	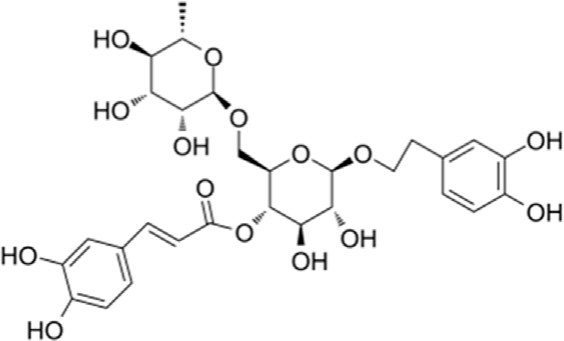	Lianqiao
Cynaroside	5373-11-5	5280637	C_21_H_20_O_11_	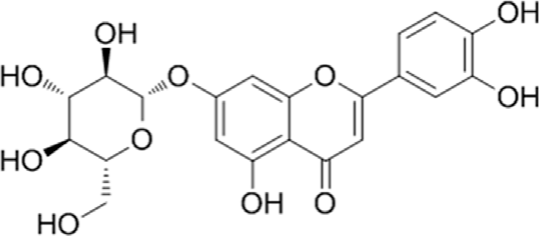	Jinyinghua
Cimifugin	37921-38-3	441960	C_16_H_18_O_6_	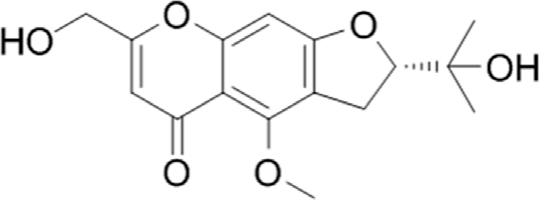	Fangfeng
5-O-Methylvisammioside	84272-85-5	21670038	C_22_H_28_O_10_	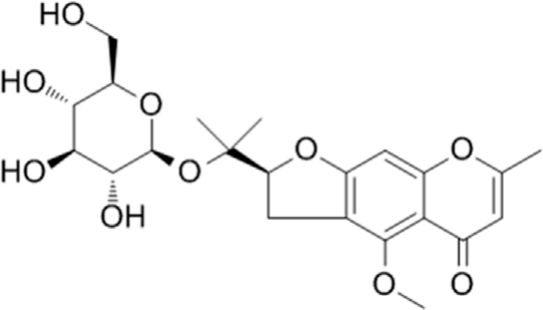	Huangqi
Calycosin 7-O-glucoside	20633-67-4	5318267	C_22_H_22_O_10_	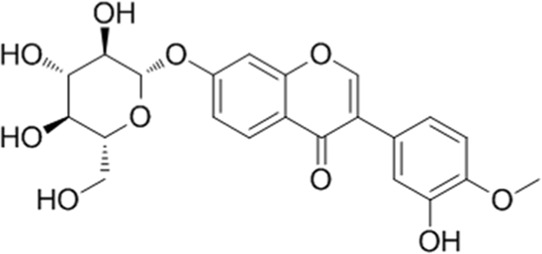	Huangqi
Ononin	486-62-4	442813	C_22_H_22_O_9_	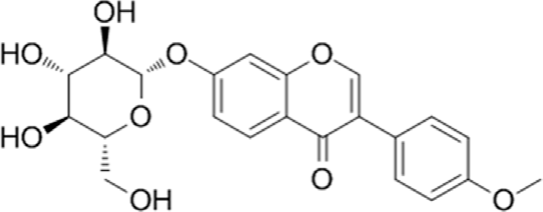	Huangqi

### YQQRG inhibited PI3K/AKT signaling pathway in UUO mice

**FIGURE 4:**
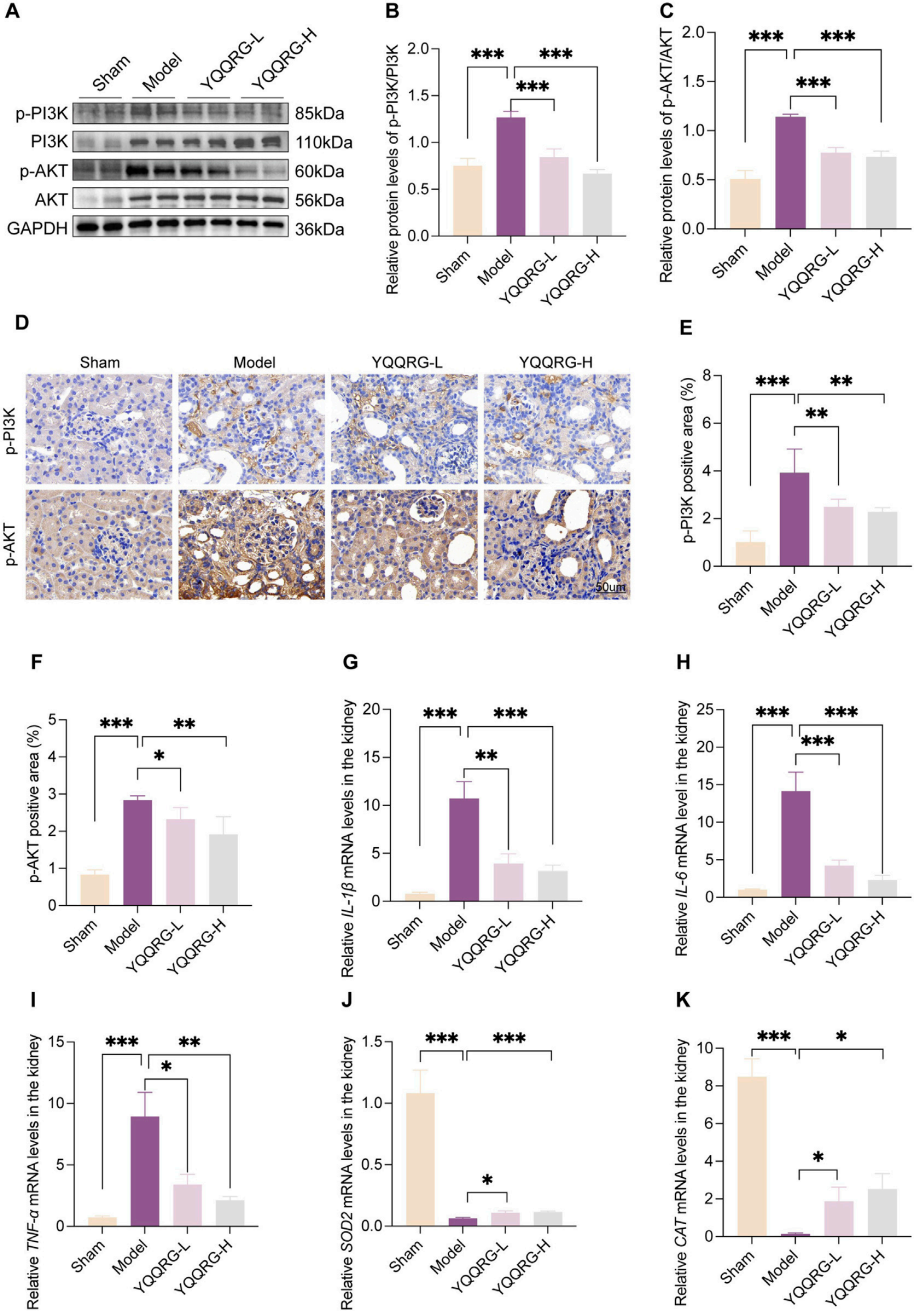
YQQRG inhibited PI3K/AKT signaling pathway in UUO mice. **(A)** Representative Western blot of p-PI3K, PI3K, p-AKT, AKT in each group. **(B)** Relative expression of p-PI3K/PI3K. **(C)** Relative expression of p-AKT/AKT. **(D)** Representative images of IHC of p-PI3K, p-AKT in each group. **(E)** p-PI3K positive area. **(F)** p-AKT positive area. **(G)** The relative mRNA levels of *IL-1*β. **(H)** The relative mRNA levels of *IL-6*. **(I)** The relative mRNA levels of *TNF-ɑ*. **(J)** The relative mRNA levels of *SOD2*. **(K)** The relative mRNA levels of *CAT*.

To investigate the role of the PI3K/AKT signaling pathway in the therapeutic effects of YQQRG in UUO mice, we evaluated the expression of key proteins associated with this pathway. While the total protein levels of PI3K and AKT remained unchanged compared to the sham group, their phosphorylation levels were significantly elevated in UUO mice. YQQRG treatment markedly reduced the phosphorylation levels of PI3K and AKT, suggesting that its anti-fibrotic effects are mediated through modulation of the PI3K/AKT pathway (Figures 4A–F). Given the critical roles of inflammation and oxidative stress in renal fibrosis progression, we further examined the impact of YQQRG on inflammatory cytokine expression. The qRT-PCR analysis showed that YQQRG significantly reduced the mRNA levels of pro-inflammatory cytokines, including IL-1β (Figure 4G), IL-6 (Figure 4H), and TNF-α (Figure 4I). The mRNA levels of antioxidant enzymes such as superoxide dismutase-2 (SOD2) (Figure 4J) and catalase (CAT) (Figure 4K) were increased. The above results suggest that YQQRG has a dual effect of reducing inflammation and enhancing antioxidant defense in UUO mice.

#### YQQRG inhibited PI3K/AKT signaling pathway in TGF-β1 induced HK-2 cells

**FIGURE 5:**
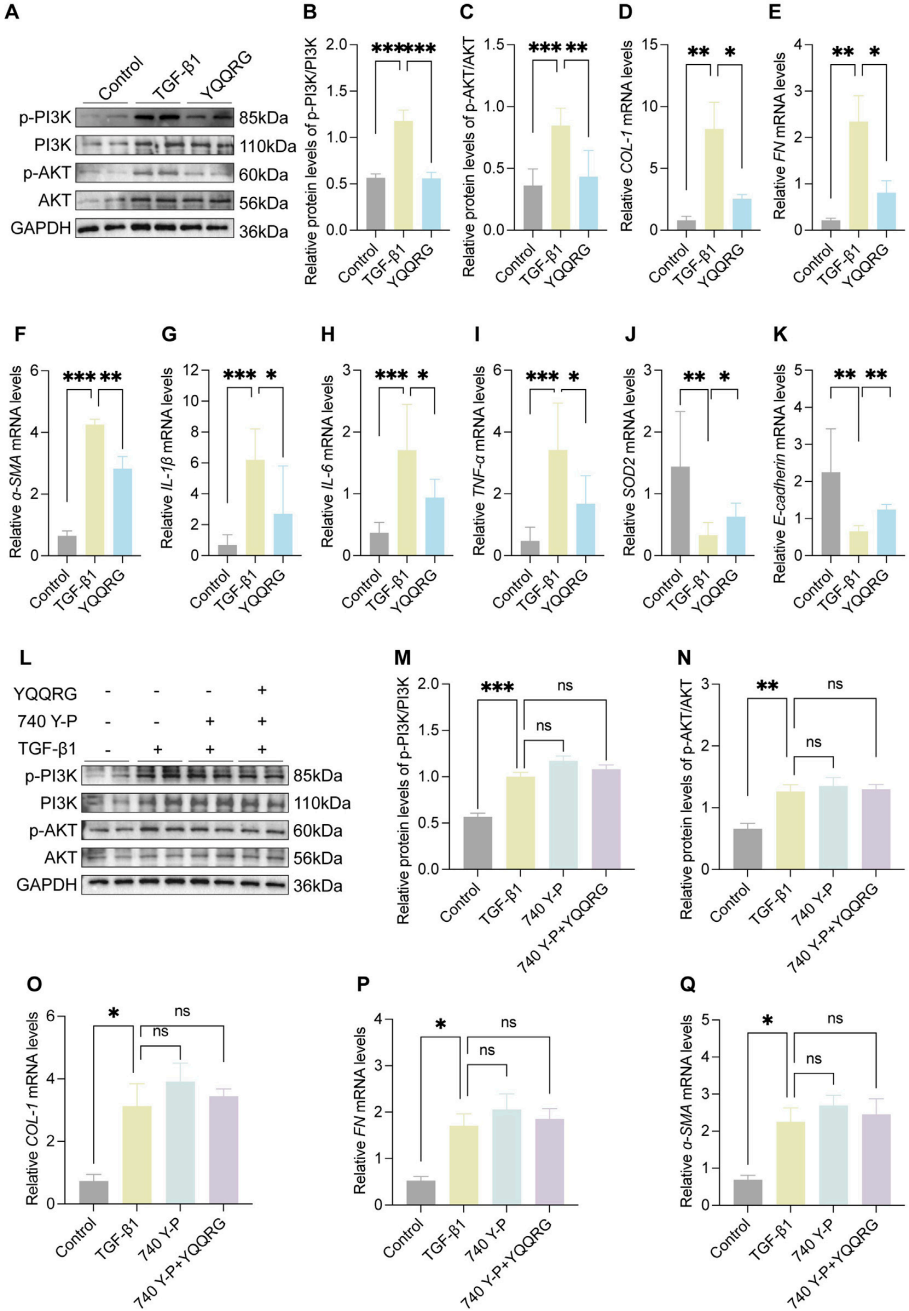
YQQRG inhibited the PI3K/AKT signaling pathway in TGF-β1 induced HK-2 cells. **(A)** Representative Western blot of p-PI3K, PI3K, p-AKT, AKT in each group (*n* = 3). **(B)** Relative expression of p-PI3K/PI3K. **(C)** Relative expression of p-AKT/AKT. **(D)** The relative mRNA levels of *COL-1*. **(E)** The relative mRNA levels of *FN*. **(F)** The relative mRNA levels of *ɑ-SMA*. **(G)** The relative mRNA levels of *IL-1*β. **(H)** The relative mRNA levels of *IL-6*. **(I)** The relative mRNA levels of *TNF-ɑ*. **(J)** The relative mRNA levels of *SOD2*. **(K)** The relative mRNA levels of *E-cadherin*. **(L)** Representative Western blot of p-PI3K, PI3K, p-AKT, AKT in each group (*n* = 3). **(M)** Relative expression of p-PI3K/PI3K. **(N)** Relative expression of p-AKT/AKT. **(O)** The relative mRNA levels of *CO-1*. **(P)** The relative mRNA levels of *FN*. **(Q)** The relative mRNA levels of *ɑ-SMA*.

To further elucidate the mechanism of action of YQQRG, we investigated its effects on TGF-β1-induced activation of the PI3K/AKT signaling pathway in HK-2 cells. TGF-β1 stimulation significantly increased the phosphorylation levels of PI3K and AKT (Figures 5A–C) and upregulated the expression of fibrotic markers, including α-SMA, FN, and COL-1 (Figures 5D–F). In addition, mRNA levels of IL-1β, IL-6, and TNF-α, were elevated, and levels of SOD2 and E-cadherin were decreased in TGF-β1-treated cells. YQQRG treatment effectively reversed these changes (Figures 5G–K), suggesting its anti-fibrotic and anti-inflammatory properties.

To confirm whether YQQRG exerts its effects through the PI3K/AKT pathway, we employed the PI3K/AKT agonist 740Y-P. The downregulation of p-PI3K, p-AKT, and fibrotic markers (α-SMA, FN, and COL-1) by YQQRG was counteracted by 740Y-P, effectively nullifying YQQRG’s protective effects against renal fibrosis (Figures 5L–Q). These *in vitro* findings are consistent with the *in vivo* results, collectively indicating that YQQRG’s anti-fibrotic effects are mediated through inhibition of the PI3K/AKT signaling pathway.

### Molecular docking analysis of bioactive components with target proteins

**FIGURE 6:**
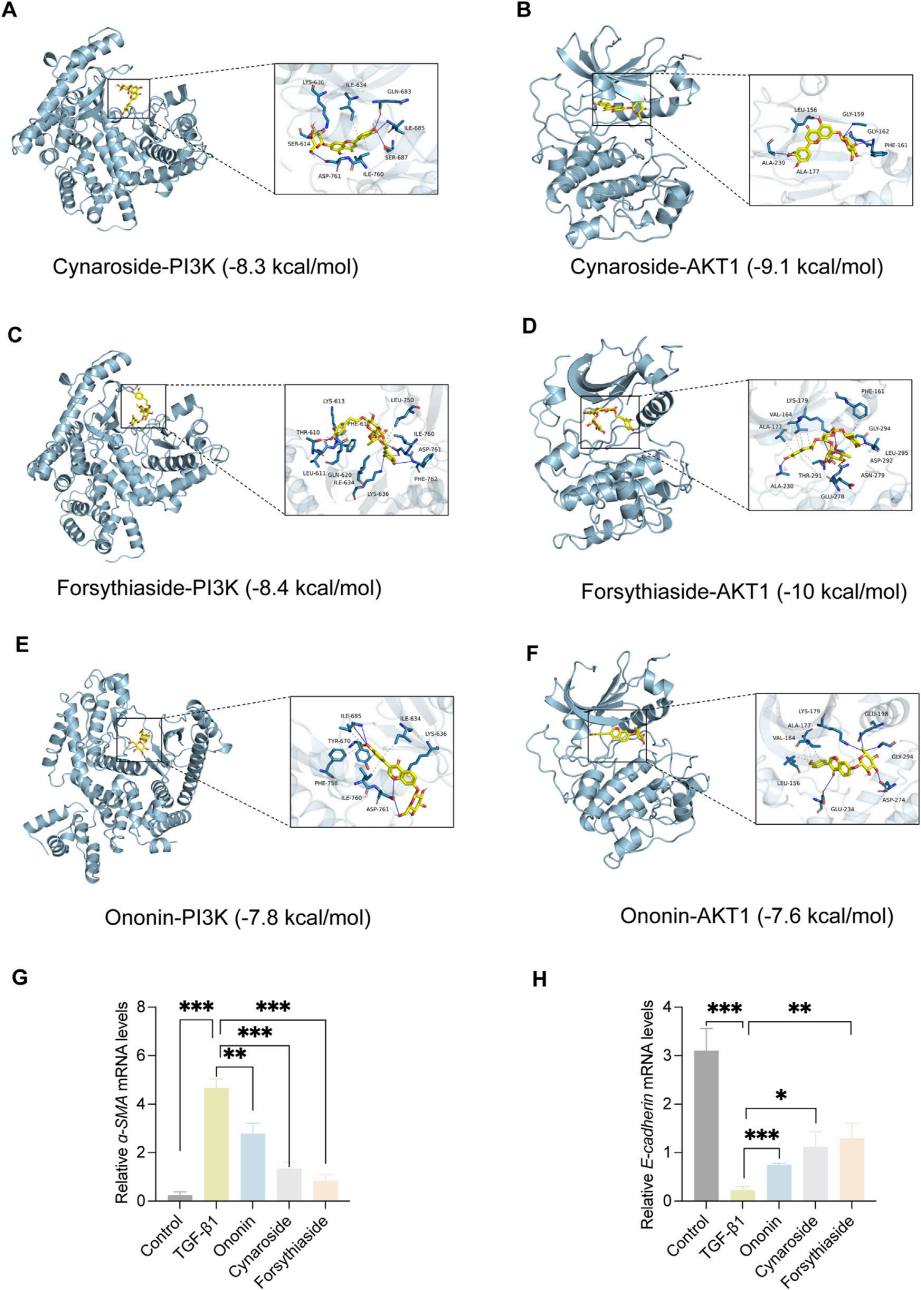
The Visualization of molecular docking. **(A)** Visualization of docking results between PI3K and Cynaroside. **(B)** Visualization of docking results between AKT1 and Cynaroside. **(C)** Visualization of docking results between PI3K and Forsythiaside. **(D)** Visualization of docking results between AKT1 and Forsythiaside. **(E)** Visualization of docking results between PI3K and Ononin. **(F)** Visualization of docking results between AKT1 and Ononin. **(G)** The relative mRNA levels of *ɑ-SMA*. **(H)** The relative mRNA levels of *E-cadherin*.

To explore the interaction of active components and targets, the molecular docking was performed. Three critical active components of YQQRG (Cynaroside, Forsythiaside, Ononin) were selected for molecular docking with the key targets PI3K and AKT1, which are essential in renal fibrosis therapy. The docking results showed good binding activity (affinity ≤5.0 kcal/mol) for the key components and targets, with Forsythiaside having the lowest binding energy to AKT1, suggesting that AKT1 has the most vital binding ability. The molecular docking results for each important compound and its selected target are shown in the figure (Figures 6A–F). The findings indicate that the docking sites and structures of small molecule ligands and protein receptors are stable. To validate the computational predictions, we performed *in vitro* experiments using TGF-β1-induced HK-2 cells treated with the three bioactive compounds. qRT-PCR analysis demonstrated that all three compounds significantly modulated fibrosis-related biomarkers and upregulated E-cadherin expression. Among the tested compounds, Forsythiaside exhibited the most pronounced anti-fibrotic effects, consistent with its superior binding affinity observed in the docking studies (Figures 6G,H). However, further mechanistic investigations are warranted to fully elucidate the underlying molecular pathways and therapeutic potential of these compounds.

## Discussion

**FIGURE 7:**
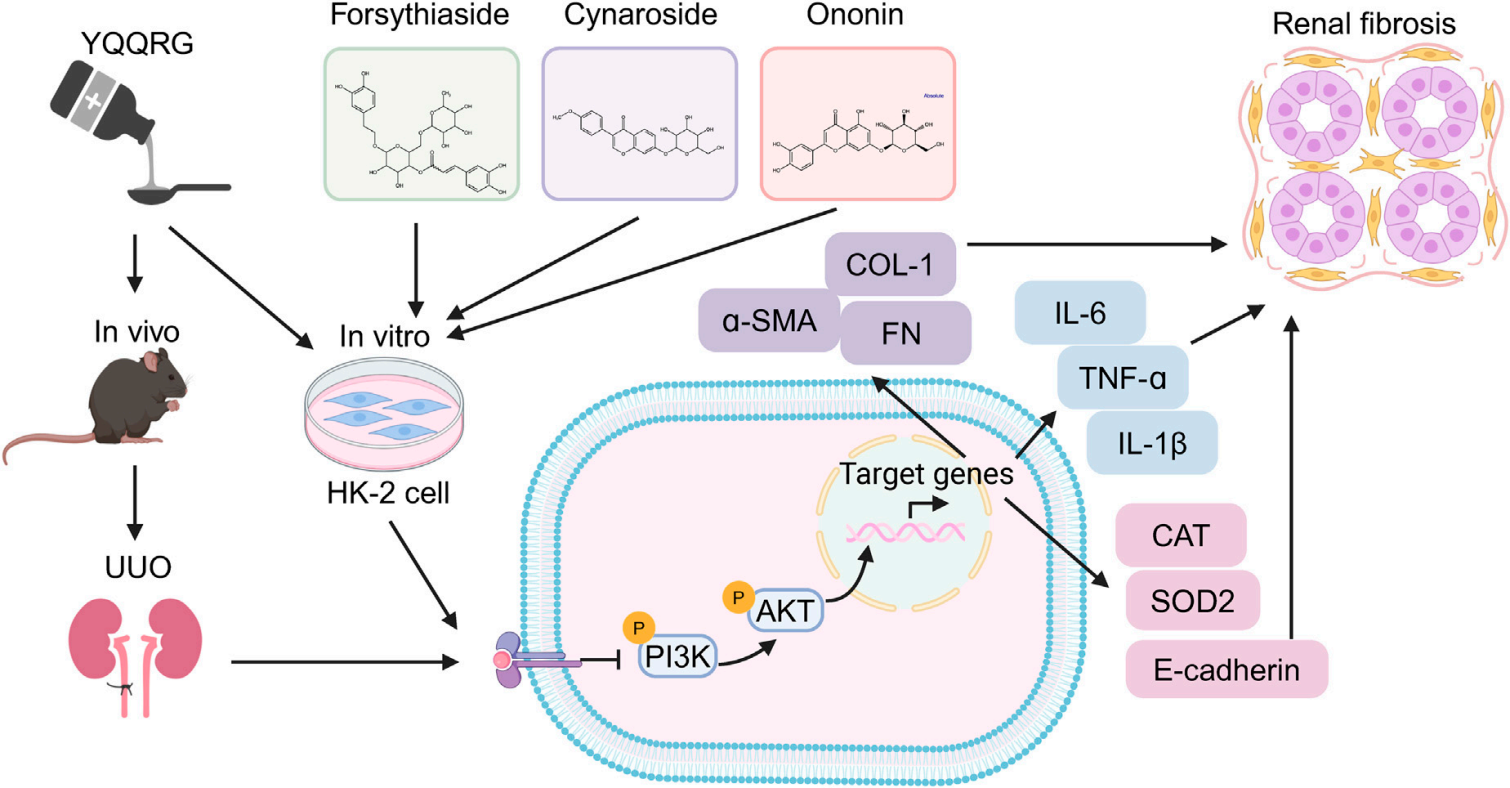
Mechanism of YQQRG in the treatment of renal fibrosis.

This study demonstrates that YQQRG significantly improves kidney function and attenuates renal fibrosis in a UUO mouse model. Through integrated network pharmacology and RNA-seq analyses, we identified the PI3K/AKT signaling pathway as the primary mechanistic target of YQQRG in UUO. Experimental validation confirmed that YQQRG suppresses PI3K/AKT activation both *in vivo* and *in vitro*. Furthermore, the use of a PI3K/AKT agonist and molecular docking studies corroborated these findings, reinforcing the central role of this pathway in YQQRG’s therapeutic effects (Figure 7).

TCM has gained increasing recognition globally for its favorable safety and efficacy profiles, warranting further clinical and basic research (Silver et al., 2021). YQQRG, a multi-herbal TCM formulation used for CKD treatment, has been clinically applied for over two decades. It effectively alleviates patient symptoms, reduces proteinuria, and slows kidney disease progression. Previous studies have demonstrated YQQRG’s ability to repair podocyte damage and ameliorate glomerulosclerosis (Wen et al., 2015). However, research on its effects on tubular injury remains limited. Our findings reveal that YQQRG significantly improves kidney function, highlighting its potential in addressing tubular injury. Renal fibrosis, the final common pathway of various CKDs, is increasingly prevalent due to aging populations and the rise of metabolic diseases (Schreibing et al., 2023). Its pathogenesis involves multiple mechanisms, including inflammation, oxidative stress, apoptosis, senescence, epithelial-mesenchymal transition (EMT), and extracellular matrix (ECM) dysregulation (Huang R. et al., 2023). Our study showed that YQQRG significantly reduced the expression of fibrosis markers.

The clinical efficacy of TCM formulas depends largely on their chemical composition (Gu et al., 2024). Recent advances in MS technology have thoroughly enhanced the identification of drug constituents, and LC/MS, which combines high-performance liquid chromatography and MS, has become a powerful tool for pharmacological studies (Liang et al., 2024). In our previous study, LC/MS identified 24 chemical constituents in YQQRG, of which 10 were confirmed to be blood-entering compounds. Combined with network pharmacological analysis of these 10 blood-entering compounds, 403 potential targets were obtained, with the PI3K/AKT pathway occupying a prominent position. RNA-seq analysis of fibrotic renal tissues significantly ranked PI3K/AKT at the top of the list, consistent with the results of the YQQRG target analysis. The PI3K/AKT pathway is closely associated with fibroblast proliferation (Fang et al., 2020), EMT (Tuoheti et al., 2024) and ECM deposition (Jia et al., 2025), which are key processes in fibrotic kidney disease. Specifically, PI3K/AKT signaling enhances TGF-β1-induced fibrogenesis and inhibits apoptosis of fibrotic cells, leading to renal tissue scarring and functional decline (Yang et al., 2024). PI3K, a central signal-transducing enzyme, regulates diverse cellular processes, including proliferation, survival, adhesion, differentiation, and cytoskeletal organization. It is activated by various receptors, such as tyrosine kinase receptors, G-protein-coupled receptors, cytokine receptors, and Ras-associated GDPase receptors. AKT, a downstream effector of PI3K, plays a critical role in modulating cell migration, proliferation, and apoptosis (Glaviano et al., 2023). Various evidence suggests that the PI3K/AKT pathway is implicated in the pathogenesis of several renal diseases (Xu et al., 2021; Wang et al., 2024b), such as regulates autophagy and apoptosis, ameliorating kidney injury in diabetic nephropathy (Wang et al., 2022). Moreover, PI3K/AKT acts upstream of NF-κB, contributing to NF-κB nuclear translocation and production of pro-inflammatory cytokines (e.g., TNF-α, IL-1β, and IL-6) (Wang et al., 2024a).

Phosphorylation of the PI3K/AKT pathway is a critical driver of renal fibrosis. Phosphorylated AKT modulates glycogen synthase kinase-3beta activity, promoting fibroblast activation and matrix accumulation (Yu et al., 2022). Phosphorylated PI3K/AKT also activates the TGF-β pathway, induces EMT (Chen L. et al., 2023), and promotes fibrosis progression through dysregulation of lipid metabolism (Li et al., 2021). In this study, the UUO model group had elevated pPI3K/PI3K and p-AKT/AKT ratios and increased expression of fibrosis markers (α-SMA, FN and COL1) compared to sham controls, and YQQRG treatment reversed these changes. *In vitro* studies using TGF-β1-induced HK-2 cells confirmed the ability of YQQRG to downregulate PI3K/AKT pathway proteins and fibrotic markers. 740Y-P, a PI3K/AKT agonist, reversed the antifibrotic effects of YQQRG, further validating the central role of this pathway. Overall, these findings suggest that YQQRG exerts its antifibrotic effects by inhibiting the PI3K/AKT pathway.

Cynaroside, a flavonoid from Honeysuckle, has anti-inflammatory and antioxidant properties and can modulate macrophage polarisation via the Nrf2/HO-1 pathway (Feng et al., 2021). Forsythiaside, extracted from Forsythia, inhibits renal fibrosis and EMT by targeting THBS1 and inhibiting PI3K/AKT activation (Tuoheti et al., 2024). It also attenuates sepsis-induced acute kidney injury by modulating endoplasmic reticulum stress (Chen Y. et al., 2023) and ameliorates diabetic nephropathy by targeting oxidative stress in podocytes (Xu et al., 2022). Ononin, an isoflavonoid glycoside with anti-inflammatory and antiproliferative effects, reverses EMT by down-regulating EMT markers and matrix metalloproteinases (Ganesan et al., 2024; Sharma et al., 2024). Molecular docking studies revealed that Cynaroside, Forsythiaside, and Ononin have a strong binding affinity for PI3K and AKT, respectively. Moreover, all of these components were found to reduce α-SMA and increase E-cadherin mRNA levels in TGF-β1-induced HK-2 cells, and the difference in the effects of cynaroside was the most significant. However, further *in vivo* studies are needed to verify.

The following limitations still exist in this study: firstly, although the UUO model provides an important basis for the study of the antifibrotic mechanism of YQQRG, its pathological characteristics differ from those of human CKD, and its mechanism of action and efficacy need to be further verified in a variety of pathological models, such as the diabetic nephropathy model and ischemia-reperfusion injury model, in the future. Secondly, although the main active components of YQQRG were preliminarily screened in this study, *in vivo* pharmacokinetic studies of its components, including the determination of bioavailability, tissue distribution, metabolic pathways, and other key parameters, need to be conducted in the future. Finally, data from preclinical studies confirmed that YQQRG has significant antifibrotic effects, but its clinical efficacy and safety still need to be tested in multicentre, large-sample, randomized controlled clinical trials. These studies will provide important experimental basis and theoretical support for the future clinical translation of YQQRG.

## Conclusion

YQQRG demonstrates significant potential as a therapeutic agent for mitigating renal fibrosis. Comprehensive *in vivo* and *in vitro* studies evidence that the primary mechanism underlying its efficacy involves the inhibition of the PI3K/AKT signaling pathway. This research establishes a robust experimental foundation for the clinical application of YQQRG in CKD treatment.

## Data Availability

The original contributions presented in the study are included in the article/Supplementary Material, further inquiries can be directed to the corresponding authors.

## Abbreviations

ɑ-SMA, alpha-smooth muscle actin; AKT, protein kinase B; ALB, albumin; ALT, alanine aminotransferase; AST, aspartate aminotransferase; BUN, blood urea nitrogen; CAT, catalase; CKD, chronic kidney diseases; COL-1, collagen type I; ECM, extracellular matrix; EMT, epithelial-mesenchymal transdifferentiation; FN, fibronectin; HE, hematoxylin and eosin; HK-2, human renal proximal tubular epithelial; IHC, immunohistochemistry; IL-1β, interleukin-1 beta; KEGG, Kyoto Encyclopedia of Genes and Genomes; LC/MS, liquid chromatography-mass spectrometry; NF-κB, nuclear factor-kappaB; PI3K, phosphatidylinositol 3-kinase; qRT-PCR, quantitative real-time PCR; RNA-Seq, RNA sequencing; SCr, serum creatinine; SOD2, superoxide dismutase-2; TCM, Traditional Chinese Medicine; TGF-β1, Transforming growth factor-beta1; TNF-ɑ, tumor necrosis factor-alpha; UUO, unilateral ureteral obstruction; YQQRG, Yiqi Qingre Gao.
