# Supplementary figures and images for: Yiqi Qingre Gao alleviates renal fibrosis in UUO mice via PI3K/AKT pathway
Source: Front Pharmacol. 2025 Mar 24;16:1538061. doi: 10.3389/fphar.2025.1538061 (PMC11973907; doi:10.3389/fphar.2025.1538061)

# CKO-UU0-vs-CKO: q-value<0.05 && |log<sub>2</sub> FC|>1

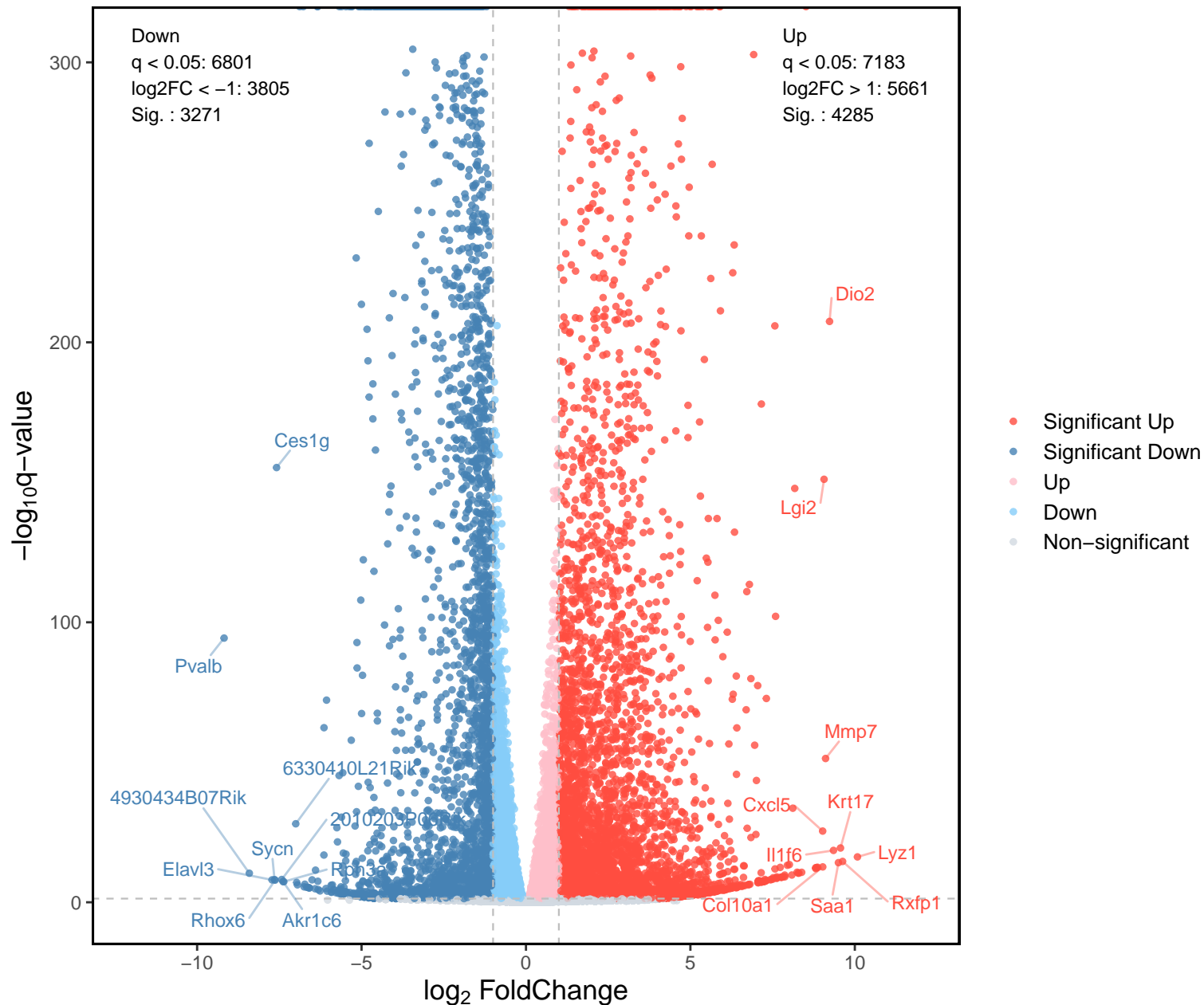

Supplement: Supplementary file 1 [file Supplementaryfile1.zip › Supplementary figure 2.PDF]

# CKO-UU0-vs-CKO: q-value<0.05 && |log<sub>2</sub> FC|>1

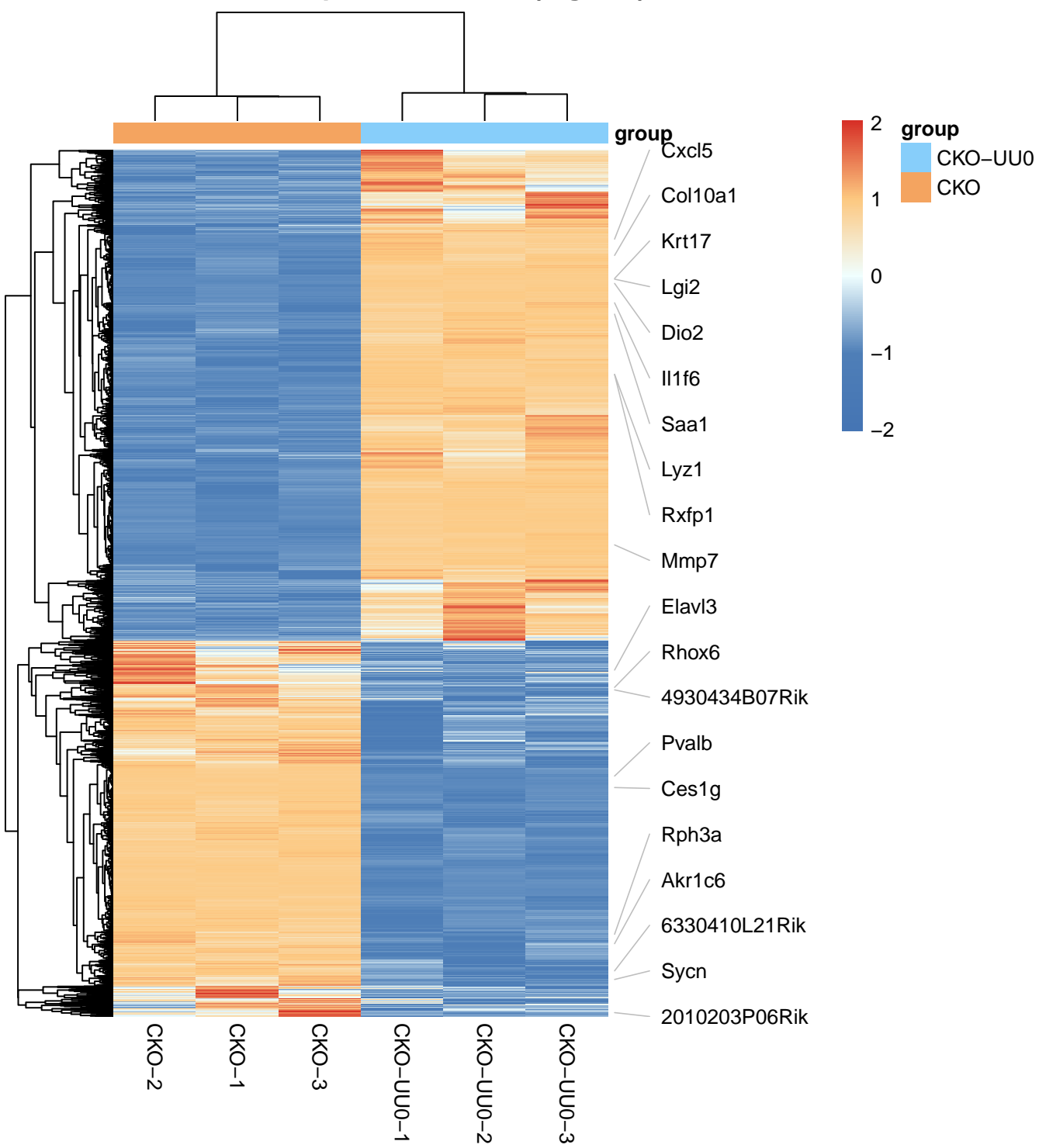

Supplement: Supplementary file 1 [file Supplementaryfile1.zip › Supplementary figure 1.PDF]

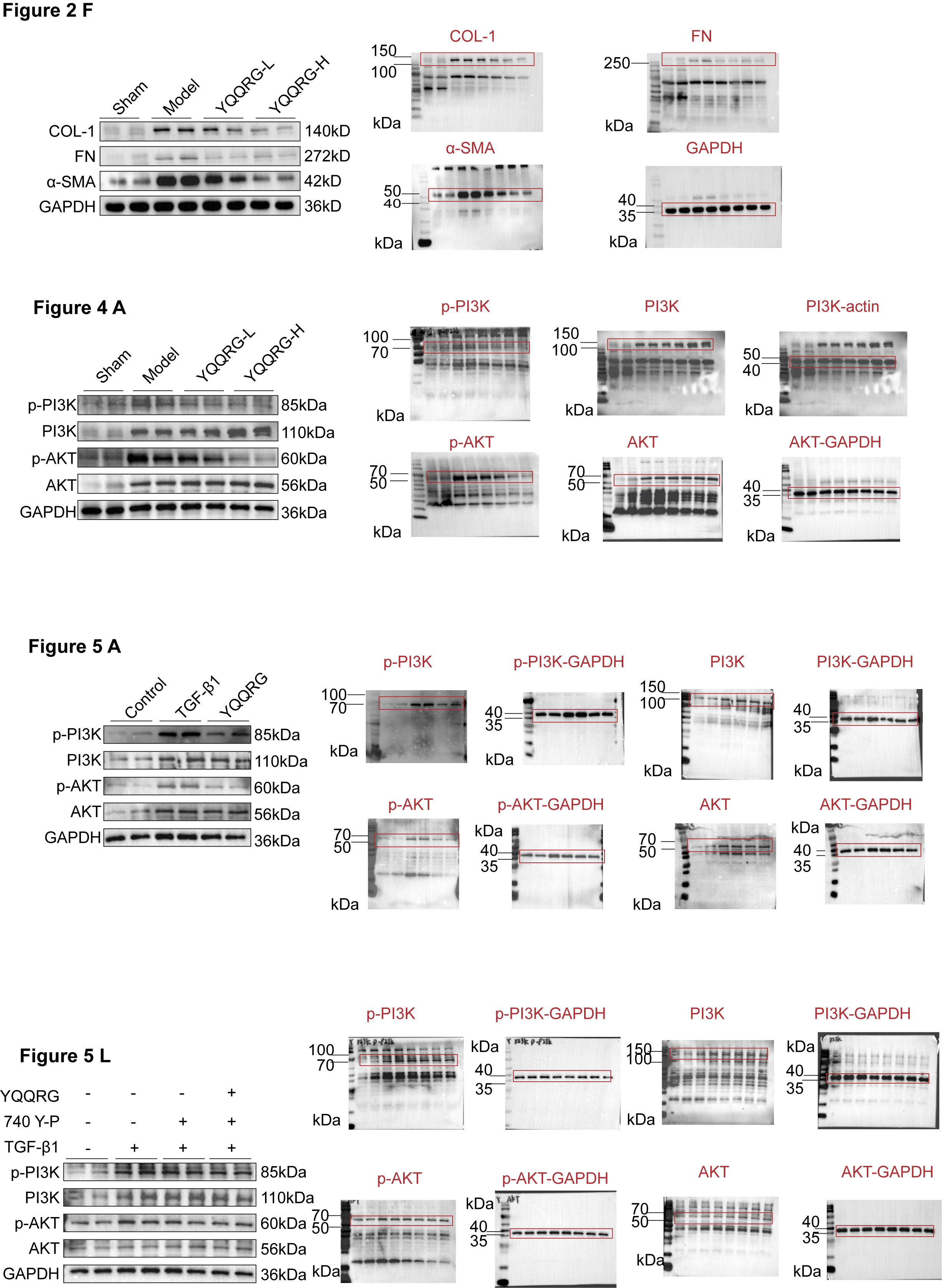

Supplement: Supplementary file 1 [file Supplementaryfile1.zip › Supplementary figure 3.TIF]
